# Primary Hypoparathyroidism Mimicking Ankylosing Spondylitis in a Young Man with Fahr's Syndrome: A Case Report

**DOI:** 10.7759/cureus.10426

**Published:** 2020-09-13

**Authors:** Sreethish Sasi, Ali Rahil, Surjith Vattoth, Priyanka Cackamvalli, Wafa Abdullah

**Affiliations:** 1 Internal Medicine, Hamad Medical Corporation, Doha, QAT; 2 Radiology/Neuroradiology, University of Arkansas for Medical Sciences, Little Rock, USA; 3 Rheumatology, Hamad Medical Corporation, Doha, QAT

**Keywords:** hypoparathyroidism, spondyloarthropathies, hypocalcemia, fahr’s syndrome

## Abstract

Patients with chronic idiopathic hypoparathyroidism may develop neurological complications, including calcification of the basal ganglia and other areas of the brain. In Fahr's syndrome, intracranial calcification is associated with an underlying disorder such as hypo or hyperparathyroidism. We report the case of a 37-year-old gentleman, with a history of bilateral cataract surgery and seizures, who presented with a new episode of seizure and was found to have severe hypocalcemia and bilateral symmetric intracranial calcification due to previously diagnosed primary hypoparathyroidism. He had symptoms and signs mimicking ankylosing spondylitis (AS), but with negative radiological and serological findings, not fitting into the diagnosis of axial spondyloarthropathies (SpA), as per standard criteria. Patients with long-standing idiopathic hypoparathyroidism can have severe calcification of soft tissues and bones, including vertebrae and paravertebral soft tissues, causing inflammatory back pain and stiffness. It is vital to report such cases as their occurrence is rare, and physicians should be aware of the possibility while evaluating patients with inflammatory back pain. Treatment in these cases is directed towards hypocalcemia and underlying primary pathology rather than spondyloarthropathy.

## Introduction

Critical initial laboratory evaluations for patients with calcium derangements are phosphorous, magnesium, intact parathyroid hormone (PTH), and vitamin D [1]. Hypocalcemia associated with low levels of PTH is due to primary hypoparathyroidism, with a wide range of etiologies such as iatrogenic (radio-iodine ablation or thyroidectomy), infiltrative diseases, congenital syndromes, mitochondrial gene defects, neonatal, auto-immune and idiopathic [2]. Patients with chronic idiopathic hypoparathyroidism may develop neurological complications, including intracranial calcifications [3]. Fahr's disease and Fahr's syndrome are two conditions characterized by calcification in the brain resulting in neurological or psychiatric sequelae. Fahr's disease is a congenital (autosomal dominant or recessive) disorder with an age of onset from 40 to 60 years. Fahr's syndrome presents between 30 and 40 years of age, and the bilateral intracranial calcification is associated with an underlying disorder such as idiopathic hypoparathyroidism, secondary hypoparathyroidism, pseudohypoparathyroidism, or hyperparathyroidism. Treatment of Fahr's disease is only symptomatic as there is no specific remediation, whereas, for Fahr’s syndrome, it is directed at the particular pathology [4]. As PTH is the principal regulator of calcium and phosphate metabolism, hypoparathyroidism causes hyperphosphatemia to maintain ionic products of calcium and phosphate in the normal range. This, in turn, results in ectopic soft tissue calcifications [5]. As per the Assessment of SpondyloArthritis International Society (ASAS) criteria, inflammatory back pain should have at least four out of the following five features: (a) insidious onset, (b) pain at night with improvement upon getting up, (c) age at onset less than 40 years, (d) improvement with exercise, and (e) lack of improvement with rest [6].

## Case presentation

A 37-year-old Indian gentleman was brought to the emergency department (ED), in a post-ictal state, after he was witnessed to have an episode of generalized tonic-clonic seizures, one hour back. He was lying down when he suddenly had jerky movements, which began on his upper limbs and later involved the whole body. The episode lasted three minutes and was associated with loss of consciousness and tongue bite, with no fecal or urinary incontinence. He did not have any trauma or emotional events before the onset of the seizure. His history was significant for a similar episode four years back, after which he was started on treatment with oral levetiracetam 250 milligrams twice daily. He had severe hypocalcemia at presentation and was started on calcium and vitamin D supplementation. He stopped levetiracetam after a year of usage, but continued calcium and vitamin D supplements. He moved to Doha two years back to work as a plumber and did not have any clinical follow-up data since then. Calcium and vitamin D supplements were missed for a week before the current event, as he ran out of stock. His history was also significant for inflammatory lower back pain (as per ASAS criteria mentioned above) for the last four years. He had substantial morning stiffness, difficulty in waking up from lying position, and moving, improving by the end of the day, but not responding to rest or nonsteroidal anti-inflammatory drugs. He does not use cigarettes, alcohol, or illicit drugs, and there is no family history of seizures or spondyloarthropathies. He does not remember any episodes of uveitis, enthesitis, psoriasis, dactylitis, or peripheral arthritis. He had a history of cataract and underwent surgery to both eyes at 30 years.

An initial evaluation in the ED showed that he was afebrile with stable vitals (oral temperature: 36.8 °Celsius, respiratory rate: 18 per minute, blood pressure: 125/84 mmHg, saturation: 98% in room air). There was erythema and bleeding on the left side of the tongue. There were no signs of head trauma. His neck was stiff with limited flexion. He was confused and disoriented during first hours of the presentation, which later resolved. Other components of neurological examination, performed after he regained full consciousness, including cranial nerves, motor, sensory, and cerebellar systems were within normal limits. After initial assessment and management, further detailed analysis from the rheumatological point of view was done two days later, after he was stabilized. He had limited flexion and rotation of his hip and neck (<10 degrees). Faber test and Schober test (11 cm) were positive, and occiput to wall distance was 2.5 cm. The chest expansion was also limited (2 cm). There was no uveitis, enthesitis, or dactylitis. Respiratory, cardiovascular, and abdominal examinations were unremarkable. 

The initial laboratory results were significant for severely low calcium (0.99 mmol/L or 3.97 mg/dL), high phosphorous (5.05 mg/dL), and low PTH (1 pg/mL). There was no leukocytosis, anemia, or a rise in inflammatory markers. The liver, kidney, thyroid functions, vitamin D levels, and other electrolytes, including magnesium, were within normal limits. 24-hour urine calcium was low (<1.7 mmol), and there was no evidence of adrenal insufficiency (normal basal plasma adrenocorticotropic hormone, and 24-hour urine free cortisol). Blood tests done to evaluate his lower back pain revealed a normal erythrocyte sedimentation rate (ESR = 10 mm/hr), C-reactive protein (CRP = 4.5 mg/L), low uric acid (115 µmol/L), negative anti-nuclear antibody, negative anti-double-stranded DNA antibody, negative rheumatoid factor, and negative human leukocyte antigen (HLA) B27. The results of his relevant laboratory tests, at initial presentation, during treatment, at discharge, and during follow-up after a week are shown in Table 1.

**Table 1:** Results of laboratory tests at presentation (1), during hospital stay (2), during discharge (3) and during follow-up after one week (4)

Detail	Result				Normal Range
	1	2	3	4	
White Blood Cells (x10^3^/µL)	6.3	6.7	-	-	4-10
Platelets (x10^3^/µL)	224	249	-	-	150 - 400
Haemoglobin (gm/dL)	13.3	13.9	-	-	13.0-17.0
Urea (mmol/L)	4.7	5.6	3.9	4.1	2.8 – 8.1
Creatinine (µmol/L)	64	68	72	82	62 – 106
Total Bilirubin (µmol/L)	14	12.8	12	14.7	3.4 – 20.5
Alkaline Phosphatase (U/L)	70	72	68	60.7	40 - 150
Alanine Aminotransferase (U/L)	7	5	7	9	0 - 55
Aspartate Aminotransferase (U/L)	23	27	22	32	5 - 34
C – Reactive Protein (mg/L)	0.5	-	4.5	-	0 - 5
Erythrocyte Sedimentation Rate, ESR (mm/hr)	-	10	-	-	2-28
Corrected Calcium (mmol/L)	0.91	2.01	1.70	1.96	2.15-2.5
Ionised Calcium (mmol/L)	-	0.96	0.78	-	1.18-1.32
Serum Phosphorous (mmol/L)	1.63	1.82	1.83	1.88	0.81-1.45
Magnesium (mmol/L)	0.76	0.89	0.71	0.75	0.66-1.07
Parathyroid hormone (PTH) (pg/mL)	1	-	-	-	15 - 65
25-hydroxycholecalciferol (ng/mL)		31			>30
Creatine Kinase, CK (U/L)	-	873	355	-	39 - 308
24-hour Urine Calcium (mmol/24Hrs)	-	<1.7	-	-	2.5-7.5
Thyroid Stimulating Hormone, TSH (mIU/L)	-	1.66	-	-	0.3-4.2
Free T4 (pmol/L)	-	18.9	-	-	11.6-21.9
Uric Acid (µmol/L)		115			202 - 416
Basal Plasma Adrenotropicocortic hormone, ACTH (pmol/L)		3.2			1.55 - 11.1
24-hour Urine free cortisol (µg/1.7 m^2^/day)		32			10 - 84

**Figure 1:**
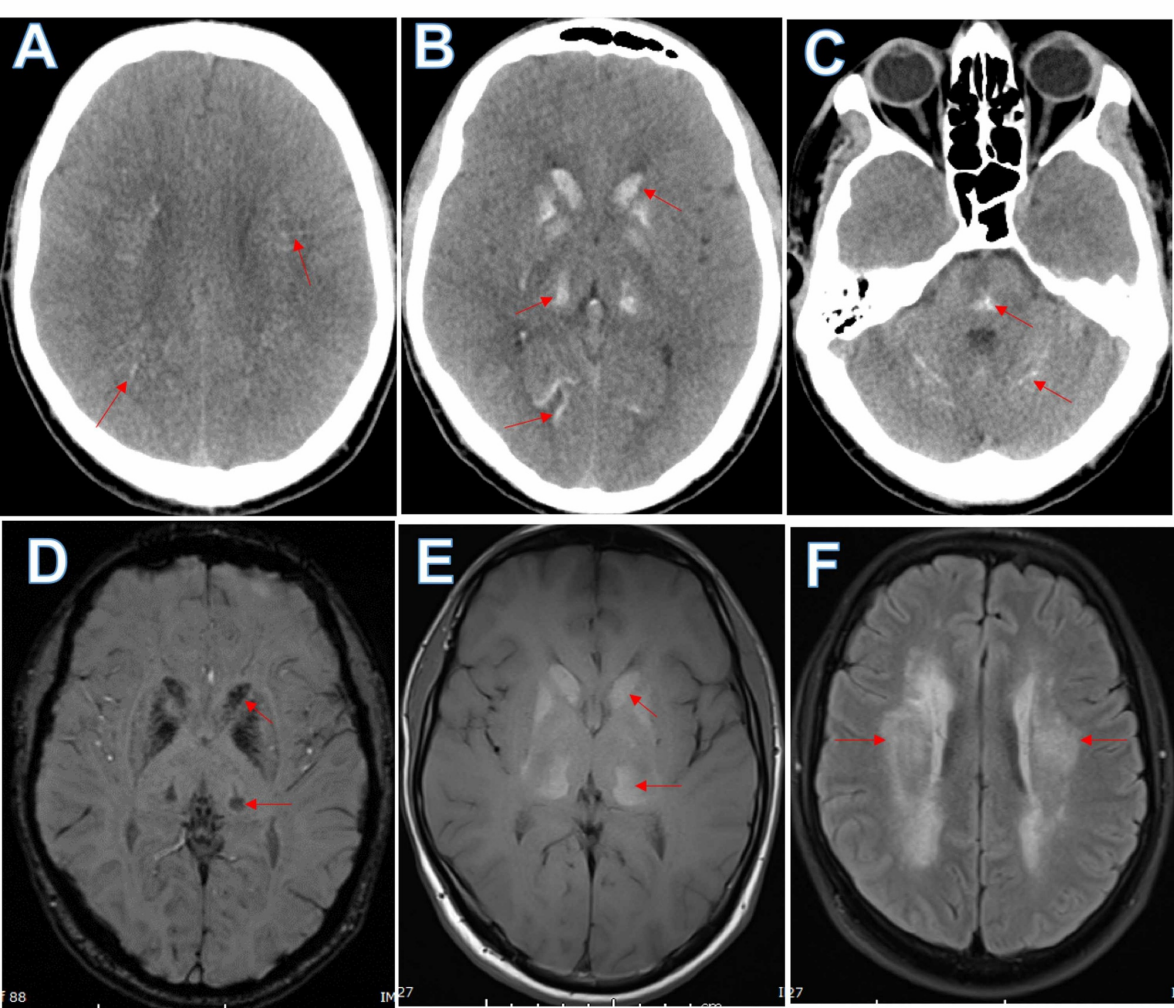
Brain imaging shows intracranial calcification Computed tomography (CT) scan of the brain shows, (A) - bilateral tiny deep white matter calcifications (arrows), (B) - prominent basal ganglia, thalami and posterior juxtacortical calcifications (arrows), and (C) - pontine, and cerebellar folia calcifications (arrows). Brain magnetic resonance imaging (MRI) demonstrates, (D) - expected blooming hypo intensity of the calcifications on susceptibility-weighted imaging (SWI), (E) - hyperintensity on T1 weighted imaging (arrows), and (F) - abnormal hyperintensity in the deep white matter on fluid-attenuated inversion recovery (FLAIR) sequence (arrows) of brain MRI, surrounding the tiny calcifications seen on the CT scan.

Initial non-contrast computed tomography (CT) of the brain showed bilateral symmetrical calcification affecting the basal ganglia, brainstem, cerebellar hemispheres, thalamus, and corona radiata, which was confirmed by a magnetic resonance imaging (MRI) of the brain done two days later (Figure 1).

**Figure 2:**
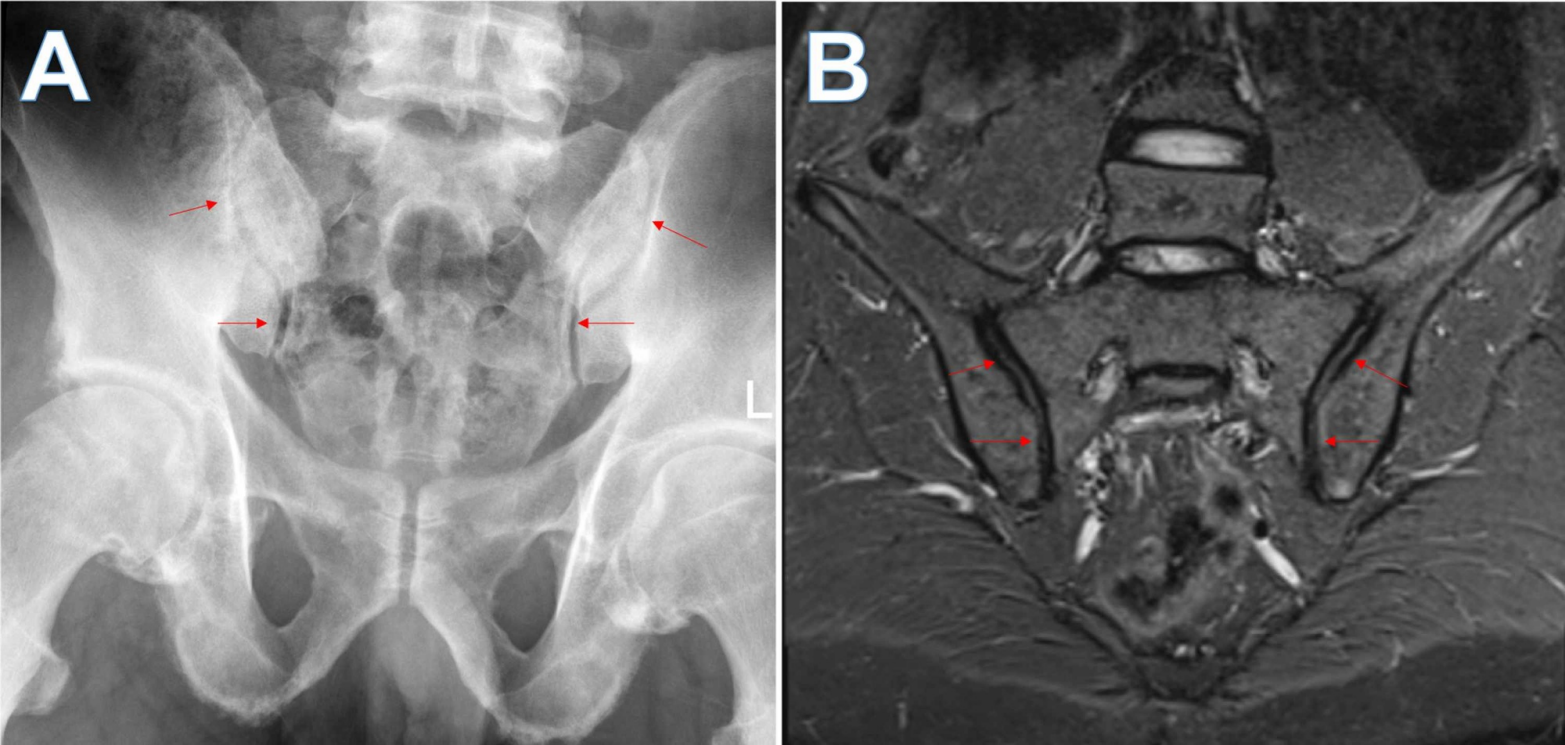
(A) Radiograph and (B) coronal T2 weighted fat suppressed inversion recovery MRI shows normal sacroiliac joints bilaterally (arrows)

X-ray of his hip showed bilateral degenerative changes with spur formation involving the superior lateral aspect of the acetabulum and relative joint space narrowing, more evident on the right side. X-ray and MRI of the sacroiliac joint were unremarkable and showed no evidence of active sacroiliitis (Figure 2).

**Figure 3:**
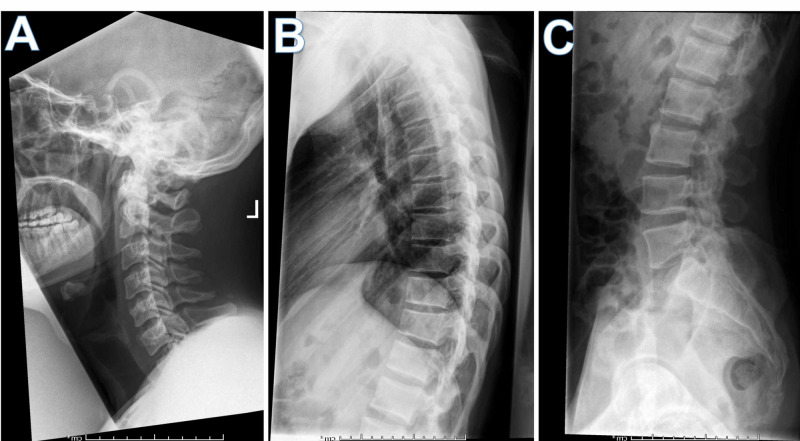
Lateral radiographs of the (A) cervical, (B) thoracic and (C) lumbar spine show no evidence of syndesmophytes, bamboo spine, or ossification of spinal ligaments, joints, or discs excluding ankylosing spondylitis

The spinal X-ray of our patient was relatively normal except for early mild spondylotic changes in cervical and lumbar regions, and relative narrowing of C4-C5, C5-C6, and L5-S1 disc spaces. There were no definite syndesmophytes, bamboo spines, or ossification of spinal ligaments, joints, or discs, as seen in patients with ankylosing spondylitis (AS) (Figure 3). 

**Figure 4:**
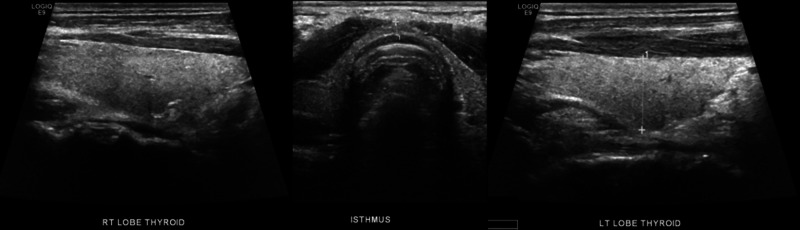
Ultrasound images showing no parathyroid lesions and a small nodule in the left lobe of an otherwise unremarkable thyroid gland

Ultrasound evaluation of the thyroid and parathyroid glands were normal except for a small left thyroid nodule. No parathyroid lesion could be detected (Figure 4).

The initial electrocardiogram showed a QTc interval of 537 milliseconds, and he was started intravenous infusion of calcium gluconate after a bolus dose. Simultaneously, he was started on oral calcium carbonate 2 grams and calcitriol 0.5 micrograms daily, which were increased to 5 grams and 2 micrograms, respectively, over the next four days, and discharged home on the same dose.

## Discussion

Low calcium, high phosphorous, and low PTH confirmed the diagnosis of primary hypoparathyroidism [1]. The patient did not have a history of surgical excision of thyroid or radiation therapy. Neither did he have any family history of auto-immune disorders, nor any features of congenital disorders. Autoimmune adrenal or thyroid involvement was also ruled out by serological tests (normal basal plasma ACTH, 24-hour urine free cortisol, and thyroid function tests) because any evidence of autoimmune involvement of other endocrine glands would be suggestive of autoimmune hypoparathyroidism. As there were no other possible etiologies identified, his hypoparathyroidism was considered as idiopathic. Our patient's idiopathic hypoparathyroidism was associated with bilateral symmetrical intracranial calcification suggesting Fahr's syndrome. Patients with Fahr's syndrome may have neurological features such as seizures, myoclonus, dystonia, and Parkinsonism. Fifty-seven percent present with movement disorders, of which Parkinsonism is the most prevalent [7]. Studies by Hempel et al. [8] and Lopez-Villegas et al. [9] had shown that clinical presentation in Fahr's syndrome depends upon the area of brain involvement. In our patient seizures were due to Fahr’s syndrome rather than hypocalcemia as other features of acute hypocalcemia were absent in him.

The onset of inflammatory back pain at a young age and the clinical examination findings of axial arthropathy pointed towards the possibility of AS. However, his negative HLA B27, absence of sacroiliitis on imaging, normal CRP and ESR, and lack of response to NSAIDs were all against the diagnosis of axial spondyloarthropathies (SpA). He did not meet the ASAS criteria for the diagnosis of axial SpA (Table 2).

**Table 2:** Assessment of SpondyloArthritis International Society (ASAS) criteria for axial spondyloarthropathies (SpA) with reference to our patient The diagnosis of axial SpA needs sacroiliitis with one other feature or positive human leukocyte antigen (HLA) B27 with two other features in patients with back-pain for more than three months and age less than 45 years [10]. *Inflammatory back pain should have at least four out of the following five features: (a) insidious onset, (b) pain at night with improvement upon getting up, (c) age at onset less than 40 years, (d) improvement with exercise, and (e) lack of improvement with rest [6].

Back-pain for more than three months with age less than 45 years			Yes
Sacroiliitis on imaging	No	Psoriasis	No
HLA B27	No	Good response to NSAIDs	No
Inflammatory back pain*	Yes	Arthritis	No
Dactylitis	No	Elevated C reactive protein	No
Inflammatory bowel disease	No	Uveitis	No
Family history of spondyloarthropathy	No	Enthesitis	No

The clinical picture of our patient closely mimics AS, but the pathological mechanism is different, as evidenced by the absence of active sacroiliitis and negative serological markers, and hence the treatment should be different. There are rare case reports of hypoparathyroidism causing rotator cuff tendonitis [11] and acute calcific epicondylitis [12], and rarely these calcifications can involve vertebrae and paravertebral soft tissues. According to Goswami et al., inflammatory back pain in patients with hypoparathyroidism may be associated with features such as calcification at the acetabular margin of the hip, preserved bone density, lack of HLA-B27 association, and equal distribution in both sexes. Mild sacroiliitis and syndesmophytes at the thoracic or thoracolumbar spine may also be present in these patients [13]. Goswami et al. also noted that spondyloarthropathy like symptoms is more common in patients with basal ganglia calcification, as in our patient. We believe that the cataract in our patient was secondary to calcification of both lenses due to severe hypoparathyroidism, as there is no other evident cause or family history. Calcification of deep white matter of the brain, lenses, and vertebral soft tissues was evident in our patient, with the primary pathology being the alteration in calcium and phosphorous metabolism due to idiopathic hypoparathyroidism [14,15].

## Conclusions

Patients with longstanding severe idiopathic hypoparathyroidism can have diffuse calcifications that involve brain, soft tissues, and bones including vertebrae and paravertebral soft tissues, causing inflammatory back pain. As the hypocalcemia is chronic, the patients may present with spondyloarthropathy with or without clinical features of hypocalcemia. The treatment in these patients should be directed towards the primary pathology rather than the spondyloarthropathy. We recommend that hypoparathyroidism be considered among the differential diagnoses in patients presenting with inflammatory back pain, and calcium, phosphorous, PTH intact, and vitamin D may be included in the initial work-up.
